# Editorial: Neuropathology of COVID-19

**DOI:** 10.3389/fneur.2023.1273866

**Published:** 2023-08-31

**Authors:** Liisa Myllykangas

**Affiliations:** Department of Pathology, University of Helsinki and HUS Diagnostic Center, Helsinki University Hospital, Helsinki, Finland

**Keywords:** COVID-19, SARS-CoV-2, neuropathology, immune mechanism, microglia, pediatric

Currently, there are more than 375,000 articles in Pubmed covering COVID-19 and SARS-CoV-2, but very few of them have been focused on COVID-19- associated neuropathology. These neuropathological studies have been mostly based on relatively small cohort sizes that have described variable tissue changes, mainly focusing on severe adult cases. Although they have formed the basis for our understanding of COVID-19-associated neuropathology, many important knowledge gaps still exist. For example, it is still unclear if the neuropathological changes are caused by direct invasion of the virus into the brain, or rather represent indirect consequences of systemic disease. The immune-mediated mechanisms and in particular the role of activated microglial cells in acute COVID-19, or in post COVID syndrome are still not established. Furthermore, there is very little published data on COVID-19-associated neuropathological findings in children and neuropathological changes associated with post COVID syndrome.

To address some of these questions this Research Topic collected four interesting papers on COVID-19 associated neuropathology.

The mini-review by Dunai et al. gives an overview of immune-mediated mechanisms for COVID-19 neuropathology. The authors highlight two important issues. First, based on recent literature, a consensus is evolving that SARS-CoV-2 does not invade the brain in most cases, suggesting that COVID-19- associated neurological problems arise from systemic indirect effects. Second, the immune system plays an important role in controlling the virus and its dysregulation may be a cause of the pathology associated with COVID-19.

Stram et al. report neuropathological findings from eight pediatric cases with SARS-CoV-2 infection in a forensic setting. Interestingly, they use a novel tool, *ex vivo* imaging, in analysis of brain microvasculature in their post-mortem studies. Their results suggest that in infants, SARS-CoV-2 infection may present as sudden unexpected infant death. Older children may suffer from severe disease and underlying conditions (such as obesity) may predispose these patients to fatal outcomes. However, similar to adults, the neuropathological changes may be subtle, including perivascular vacuolization, gliosis, and perivascular lymphocytes. *Ex vivo* neuroimaging enhanced the detection of subtle vascular changes in their study.

Matschke et al. describe results of a case-control study of 10 COVID-19 patients and 10 age-matched controls, where microglial and astroglial responses were immunohistochemically assessed. To minimize confounding factors, patients treated in intensive care, or with any evidence of sepsis were excluded. A higher degree of microglial activation was noted in younger COVID-19 patients compared to controls, whereas no difference was seen in older patients. It is of note that the degree of astrogliosis was not associated with COVID-19 in any age group.

Stein et al., assessed microglial and macrophage activation in 17 patients who died of COVID-19 infection and five controls, including one subject with influenza. The COVID-19 patients showed a distinct pattern of microglial activation, most pronounced in the white matter with emphasis in cerebellar and brain stem areas. Severe neuropathological changes were comparable to severe influenza. Interestingly, the study also included two post-COVID-19 patients, in which the inflammatory changes were similar to acute COVID-19, but less pronounced.

Addressing some of the obvious knowledge gaps in the field, these four papers provide insights into the mechanisms of COVID-19-associated neuropathology. They indicate that neuropathological changes are mostly caused by indirect effects (in contrast to direct virus invasion into the brain), and that microglial reaction is of importance in both acute and post-COVID-19 cases. Guidelines for further studies are also pointed out. Larger neuropathological tissue collections are warranted, particularly those including pediatric and post-COVID-19 cases, and tissues from the peripheral nervous system. Traditional neuropathological methods still have many limitations and novel techniques (as exemplified by *ex vivo* imaging) and tissues collected from living patients, are needed to provide better tools for understanding the pathogenesis of COVID-19.

All the authors are acknowledged for their significant contributions toward this Research Topic. Hopefully this collection of papers will encourage further studies in this important field.

## Author contributions

LM: Writing—original draft.

